# Active surveillance in long period of total neoadjuvant therapy in rectal cancer: Early prediction of poor regression response

**DOI:** 10.3389/fonc.2022.1049228

**Published:** 2022-11-10

**Authors:** Haoyu Zhang, Ke Cao, Ganbin Li, Zhiwei Zhai, Guanghui Wei, Hao Qu, Zhenjun Wang, Jiagang Han

**Affiliations:** Department of General Surgery, Beijing Chaoyang Hosptial, Capital Medical University, Beijing, China

**Keywords:** locally advanced rectal cancer, total neoadjuvant therapy, tumor regression response, risk factors, carcinoembryonic antigen

## Abstract

**Aim:**

To analyze locally advanced rectal cancer (LARC) patients and tumor characteristics during the period of total neoadjuvant therapy (TNT) and explore the risk factors that may predict poor tumor regression in response to TNT.

**Materials and methods:**

The data of 120 LARC patients who received TNT from December 2016 and September 2019 in our hospital were retrospectively analyzed. The clinicopathological characteristics of patients with different tumor regression responses were compared. Then we divided patients into two groups according to the carcinoembryonic antigen (CEA) clearance pattern after chemoradiation to explore risk factors that might predict the tumor regression response.

**Results:**

Of 120 LARC patients, 34 (28.3%) exhibited poor regression. Stratified analysis by tumor response showed that patients with poor response to TNT were more likely to obtain elevated CEA during the course of TNT (all *P* < 0.05). For those with elevated pretreatment CEA, fewer patients with poor response obtained normal CEA after chemoradiation (13.6% vs. 72.7%, *P* < 0.001). Besides, less patients’ CEA levels in the poor response group decreased by greater than 50% after chemoradiation when compared with that in the good response group (18.2% vs. 60.6%, *P* = 0.002). Stratified analysis by CEA clearance pattern after chemoradiation showed patients who obtained an elevated pretreatment CEA and decreased by less than 50% after chemoradiation were more likely to have poor response to TNT compared to others (76.2% vs. 18.2%, *P* < 0.001). Logistic multivariate analysis revealed that cN2 (95% CI 1.553-16.448), larger tumors (95% CI 2.250-21.428) and CEA clearance pattern after chemoradiation (95% CI 1.062-66.992) were independent risk factors for poor tumor regression response.

**Conclusion:**

Approximately one-fourth of LARC patients with TNT achieved a poor regression response. Here, cN2, larger tumor size before treatment and elevated CEA levels were considered predictive features of a poor response. Active surveillance of CEA levels during the TNT course are potentially important, and CEA levels after chemoradiation might have important implications for the tumor response to TNT.

## Introduction

Colorectal cancer is known as the third most common cancer in the world, and locally advanced rectal cancer (LARC) accounts for 70% of rectal cancers (1). LARC patients are typically treated with neoadjuvant chemoradiotherapy followed by total mesorectal excision (2). Traditional neoadjuvant chemoradiotherapy has significantly reduced the local recurrence rate to 5-10% after radical surgery for rectal cancer by downstaging tumors to increase the chance of successful surgical removal (3, 4); however, the incidence of distant metastasis remains as high as 30% (5).

Under this circumstance, neoadjuvant therapy (TNT) was proposed to reduce distant metastasis. The current studies defined TNT as induction chemotherapy (chemotherapy followed by concurrent chemoradiation) or consolidation chemotherapy (systemic chemotherapy following concurrent chemoradiation) (6, 7). TNT administration is considered an alternative approach by the National Comprehensive Cancer Network guidelines for locally advanced rectal cancer patients, especially those with high-risk factors (8). A series of randomized controlled trials have demonstrated that TNT significantly increased the pathological complete regression (pCR) rate and reduced distant metastases in local advanced rectal cancer compared (9–11). In addition to better compliance with TNT than traditional neoadjuvant chemoradiotherapy, TNT significantly enhanced the effectiveness of preoperative intensity-modulated radiation therapy (IMRT) and resulted in the early eradication of micrometastases and adequate tumor regression (7, 9).

However, although tumor regression appeared in most patients following TNT, approximately 20% were resistant to TNT, which manifested as slight or no tumor regression (11, 12). These patients seemed not to benefit from increased cycles of preoperative chemotherapy and a prolonged interval between radiation and surgery during TNT. The RAPIDO trial conducted by Bahadoer et al. (10) reported a higher proportion of ypT4 tumors in TNT patients than those in the traditional neoadjuvant chemoradiotherapy group (9% vs. 6%), considering that tumors might progress for patients with nonresponding tumors in the TNT group. The retrospective cohort study conducted by Chapman et al. (13) in 2022 included 102 LARC patients following TNT treatment and revealed worse progression-free survival (PFS) in patients with incomplete regression compared with those with complete regression (*P* = 0.026). Therefore, early identification of patients who might not benefit from TNT and alterations in therapeutic approaches seemed to be essential for improving prognosis.

However, studies on risk factors suggesting tumor regression of LARC patients following TNT remain lacking (13). Chapman et al. (13) explored the clinical and molecular predictors before treatment of a complete response to TNT and found that larger tumors, p53 and SMAD4 mutations and clinically node-positive cancers predict incomplete response, demonstrating that risk factors in traditional neoadjuvant chemotherapy might also predict regression response for TNT patients. Despite this, the pretreatment indicators would be less capable of providing the tumor response to TNT, as they represent only the time point before treatment rather than the whole TNT course, which might be accompanied by constant changes in these indicators. Cheong et al. (14) found that changes in carcinoembryonic antigen (CEA) levels were prognostic markers for treatment response. For these reasons, surveillance of tumor-related indicators and their changes during long periods of TNT may predict tumor regression and help in the early identification of patients who might not benefit from TNT. In this study, we collected the clinical and pathological characteristics of LARC patients during the TNT course to further explore factors that might suggest a tumor regression response to TNT.

## Materials and methods

### Patients

**Figure 1:**
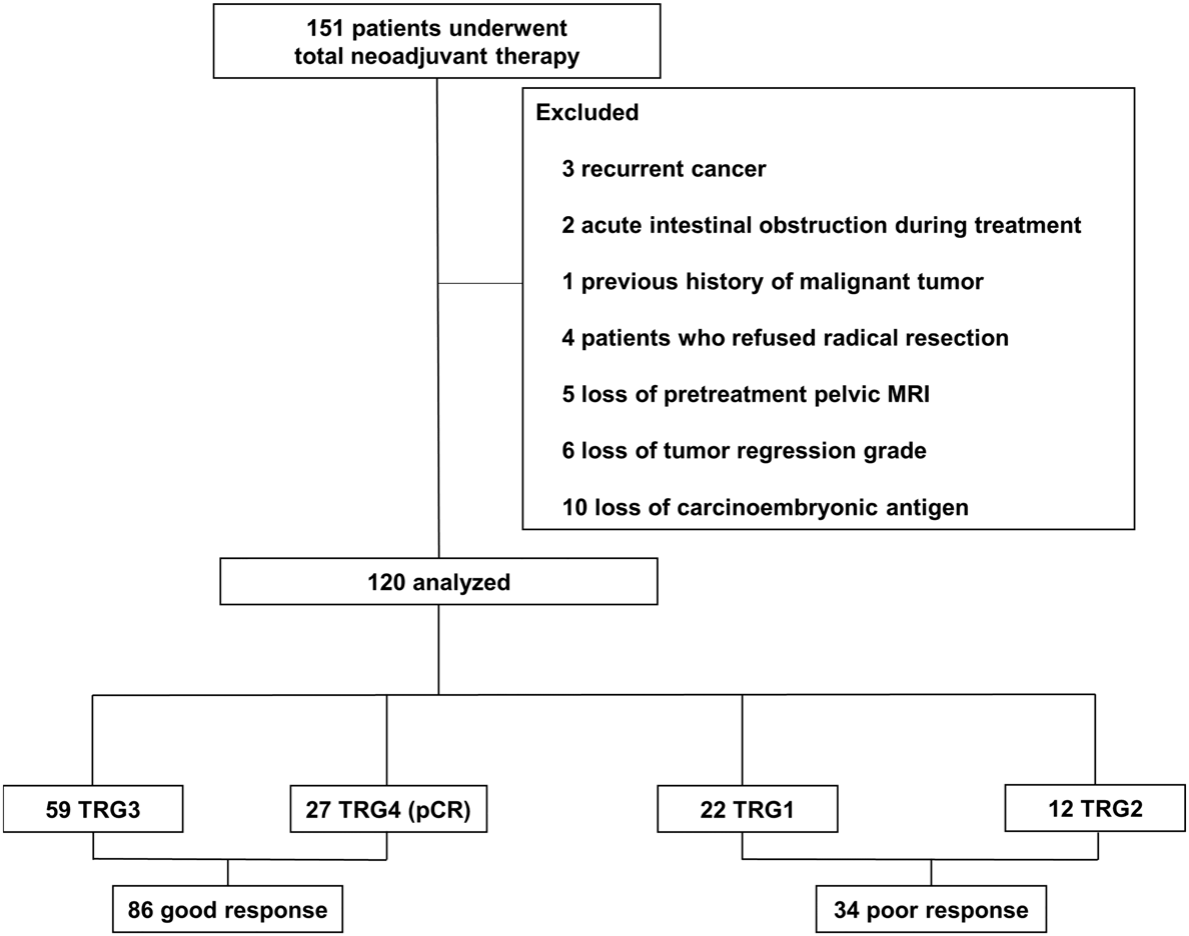
Study flow. TRG, tumor regression grade; pCR, pathological complete regression.

This study consisted of patients who underwent TNT for LARC at Beijing Chaoyang Hospital, Capital Medical University between December 2016 and September 2019. During the period, 151 LARC patients underwent TNT. The inclusion criteria were as follows: 1) distal extension less than 12 cm from the anal verge; 2) rectal adenocarcinoma determined by histology; 3) age: 18 to 80 years; 4) cT4N0 or cTxN1-2 rectal cancer with enlarged lateral nodes, extramural vascular invasion and mesorectal fascia positive based on pelvic MRI test; 5) patients who complete the whole course of TNT in our hospital; 6) American Society of Anesthesiologists (ASA) score I and II. The exclusion criteria were as follows: 1) recurrent cancer; 2) presence of unresectable cancer or acute intestinal obstruction before TNT; 3) the previous history of malignant tumor; 4) patients who refused surgery or underwent partial resection and 5) patients with missing important data (tumor regression grade, preoperative pelvic MRI and CEA). After exclusions, 120 patients were analyzed in the study (**Figure 1**). The study was conducted in accordance with the Declaration of Helsinki and was approved by the local Ethics Committee of Beijing Chaoyang Hospital (2016-science-350).

### Procedures

All patients were evaluated by coloscopy, computed tomography (CT) and pelvic MRI restaging before TNT. The patients had been selected for TNT by a multidisciplinary team (MDT). We performed TNT consisting of consolidation chemotherapy to patients. Patients were given IMRT once a day at 2.0 Gy/fraction per day, 5 days per week for a total dose of 50.0 Gy in 25 fractions, with concurrent oral capecitabine at 825 mg/m^2^ twice daily. After chemoradiation, they received two cycles of CAPOX that consisted of oxaliplatin 130 mg/m^2^ on day 1, and capecitabine 1000 mg/m^2^ twice daily on days 1 to 14, every three weeks. Pelvic MRI was performed for all patients before surgery. Radical surgery was performed at least three weeks after the completion of consolidation chemotherapy. The median interval between radiation and surgery was 12 weeks.

Radical surgery was performed in all patients regardless of the evidence of complete clinical response. Surgery was performed with total mesorectal excision and included low anterior resection (LAR), abdominoperineal resection (APR), or the Hartmann procedure depending on the distal extension from the anal verge to the tumor and intraoperative conditions.

Follow−up evaluations were scheduled every 3 months for 2 years and every 6 months thereafter. Chest X−ray, abdominal CT, and pelvic MRI were performed annually to detect local recurrence or distant metastasis. Follow-up evaluations were performed in the outpatient department and by telephone. Follow-up ended in September 2022.

### Data Collection

Clinicopathological data and patient status were obtained from the medical records database. Measurement of the distance between the lower edge of the tumor and the anus, clinical TNM staging, mesorectal fascia (MRF) and extramural vascular invasion (EMVI) before TNT were all based on preoperative MRI. CEA was recorded before treatment and after chemoradiation and each cycle of CAPOX. Adverse events (AEs) during TNT were reported based on the Common Terminology Criteria for Adverse Events version 5.0 (CTCAE) criteria. Body mass index (BMI) value was classified by applying the World Health Organization criteria.

CEA ≥ 5.0 ng/mL was considered elevated. For patients with elevated pretreatment CEA level, the clearance rate of CEA was calculated, which was considered as: (pretreatment CEA level – follow-up CEA level)/pretreatment CEA level × 100%. The system used to assess tumor regression grade (TRG) was recommended by the Dworak grading systems (15). A good response (GR) was defined as TRG 3-4, whereas TRG 1-2 was defined as a poor response (PR). pCR was defined as no residual cancer cells observed in the resection specimen, which was also regarded as TRG 4 (ypT0N0M0) (16). A positive circumferential resection margin (CRM) was defined as cancer cells detected within 1 mm of the resection margins (17).

Survival outcomes were presented by 2- and 5-year overall survival (OS) and PFS. OS was defined as the interval from the date of surgery to the date of death from any cause. PFS was defined as the interval from the date of surgery to the date of death or tumor progression, such as local recurrence or distant metastasis.

### Statistical analysis

Statistical analysis was performed with SPSS 25.0 (IBM Corp., Armonk, NY, USA) and GraphPad Prism 8 (GraphPad Software, San Diego, CA, USA). The independent t test, Fisher’s exact test, and the χ^2^ test were used to compare between-group differences. Long-term survival trends were estimated by the Kaplan−Meier method. Changes in CEA in the two study groups during the long-course of TNT were assessed by repeated-measures analysis of variance. The time trends of both groups and between-group comparisons across all time-points were shown due to no group-by-time interaction existed. Variables that had a statistically significant association at *P* < 0.05 with the regression response of patients in univariate analysis were entered into a logistic multivariable model. The area under the curve (AUC) is used as a summary measure of the receiver operating characteristics (ROC) curve and represents the discrimination ability. AUC is expressed on a scale of 0.5 to 1. The larger the AUC value, the better the classification effect. The results were reported as numbers (n) and percentages (%), means and standard deviations, or hazard ratios (HRs) with 95% confidence intervals (CIs), as appropriate, and were considered statistically significant at *P* < 0.05 in two-tailed tests.

## Results

### Baseline characteristics

These results are shown in **Table 1**.** A** total of 120 patients with stage II or III LARC who received TNT were analyzed. The average age was 59.93 years old (standard deviation: 10.33), and 94 (78.3%) were male. The average BMI was 24.29 kg/m^2^ (standard deviation: 3.43), and the average tumor diameter before treatment was 5.14 cm (standard deviation: 1.81). The most common AEs were neutropenia (12.5%), thrombocytopenia (7.5%), anemia (5.8%) and rectal pain (5.8%). No grade 4 or serious adverse events were observed in this study. Overall, 86 (71.7%) patients showed a good tumor regression response, of whom 27 (22.5%) achieved pCR. The remaining 34 (28.3%) suffered a poor regression response. The median follow-up time was 36.5 (interquartile range 13.0-60.0) months in the GR group and 25.0 (interquartile range: 11.3-56.3) months in the PR group.

**Table 1:** Clinical and pathological characteristics of patients with locally advanced rectal cancer following total neoadjuvant therapy stratified by tumor regression grade.

Characteristics	All patients	Good response (n = 86)	Poor response (n = 34)	Statistics	*P* value
Age (x ± SD), years	59.93 ± 10.33	58.89 ± 10.91	62.55 ± 8.30	t = -1.733	0.086 ^a)^
Gender (n, %)				χ^2^ = 0.033	0.857 ^b)^
Male	94 (78.3)	67 (77.9)	27 (79.4)		
Female	26 (21.7)	19 (22.1)	7 (20.6)		
BMI (x ± SD), kg/m2	24.29 ± 3.43	24.11 ± 3.33	24.75 ± 3.69	t = -0.922	0.358 ^a)^
Distance from anal verge (n, %)				χ^2^ = 0.004	0.951 ^b)^
≤ 5 cm	57 (47.5)	41 (34.2)	16 (47.1)		
> 5 cm	63 (52.5)	45 (52.3)	18 (52.9)		
Tumor diameters before treatment (x ± SD), cm	5.14 ±1.81	4.79 ± 1.57	5.54 ± 1.54	t = 2.154	0.034 ^a)^
cT stage before treatment (n, %)				χ^2^ = 0.055	0.815 ^b)^
cT2-3	90 (75.0)	64 (74.4)	26 (76.5)		
cT4	30 (25.0)	22 (25.6)	8 (23.5)		
cN stage before treatment (n, %)				χ^2^ = 5.281	0.022 ^b)^
cN0-1	44 (36.7)	37 (43.0)	7 (20.6)		
cN2	76 (63.3)	49 (57.0)	27 (79.6)		
MRF before treatment (n, %)				χ^2^ = 4.398	0.036 ^b)^
Positive	74 (61.7)	48 (55.8)	26 (76.5)		
Negative	46 (38.3)	38 (44.2)	8 (23.5)		
EMVI before treatment (n, %)				χ^2^ = 0.429	0.512 ^b)^
Positive	50 (42.3)	34 (40.5)	16 (47.1)		
Negative	68 (57.6)	50 (59.5)	18 (52.9)		
Missing	2	2	0		
CEA before treatment (n, %)				χ^2^ = 6.806	0.009 ^b)^
< 5.0 ng/ml	65 (54.2)	53 (61.6)	12 (35.3)		
≥ 5.0 ng/ml	55 (45.8)	33 (38.4)	22 (64.7)		
CEA after chemoradiation (n, %)				χ^2^ = 28.097	< 0.001 ^b)^
< 5.0 ng/ml	92 (67.7)	77 (89.5)	15 (44.1)		
≥ 5.0 ng/ml	28 (23.3)	9 (10.5)	19 (55.9)		
CEA after one cycle of CAPOX (n, %)				χ^2^ = 6.189	0.013 ^b)^
< 5.0 ng/ml	107 (89.2)	81 (94.2)	26 (76.5)		
≥ 5.0 ng/ml	13 (10.8)	5 (5.8)	8 (23.5)		
CEA after 2 cycles of CAPOX (n, %)				χ^2^ = 7.665	0.006 ^b)^
< 5.0 ng/ml	108 (90.8)	82 (95.3)	26 (76.5)		
≥ 5.0 ng/ml	12 (9.2)	4 (4.7)	8 (23.5)		
Grade 3/4 adverse events [n (%)]					
Total	29 (21.2)	19 (22.1)	10 (29.4)	χ^2^ = 0.712	0.399 ^b)^
Neutropenia	15 (12.5)	10 (11.6)	5 (14.7)	χ^2^ = 0.023	0.878 ^b)^
Anemia	7 (5.8)	3 (3.5)	4 (11.8)	χ^2^ = 1.719	0.190 ^b)^
Thrombocytopenia	9 (7.5)	6 (7.0)	3 (8.8)	χ^2^ < 0.001	1.000 ^b)^
Diarrhea	5 (4.2)	2 (2.3)	3 (8.8)	χ^2^ = 1.206	0.272 ^b)^
Vomiting	1 (0.8)	1 (1.2)	0	–	1.000 ^d)^
Radiation dermatitis	4 (3.3)	2 (2.3)	2 (5.9)	χ^2^ = 0.171	0.679 ^b)^
Rectal pain	7 (5.8)	4 (4.7)	3 (8.8)	χ^2^ = 0.199	0.655 ^b)^
Operation approaches (n, %)				χ^2^ = 1.413	0.493 ^b)^
LAR	86 (71.7)	62 (72.1)	24 (70.6)		
APR	28 (23.3)	21 (24.4)	7 (20.6)		
Hartmann	6 (5)	3 (3.5)	3 (8.8)		
Operation time (median, IQR), min	240 (180-300)	240 (180-300)	240 (192-293)	Z = -0.557	0.578 ^c)^
Blood loss (median, IQR), ml	100 (80-200)	100 (80-200)	100 (80-200)	Z = -0.578	0.628 ^c)^
Complications (n, %)					
Pelvic infection	3 (2.5)	2 (2.3)	1 (2.9)	–	1.000 ^d)^
Anastomotic bleeding	2 (1.7)	1 (1.2)	1 (2.9)	–	0.488 ^d)^
Anastomotic leakage	7 (5.8)	4 (4.7)	3 (8.8)	χ^2^ = 0.199	0.655 ^b)^
Bowel obstruction	8 (6.7)	4 (4.7)	4 (11.8)	χ^2^ = 1.003	0.317 ^b)^
ypT stage (n, %)				χ^2^ = 30.834	< 0.001 ^b)^
ypT0~2	69 (57.5)	63 (73.3)	6 (17.6)		
ypT3~4	51 (42.5)	23 (26.7)	28 (82.4)		
ypN stage (n, %)				χ^2^ = 34.003	< 0.001 ^b)^
ypN0	85 (70.8)	74 (86.0)	11 (32.4)		
ypN1~2	35 (29.2)	12 (14.0)	23 (67.6)		
Lymph nodes harvested (n, %)				χ^2^ = 2.562	0.109 ^b)^
≥ 12	71 (59.2)	47 (54.7)	24 (70.6)		
< 12	49 (40.8)	39 (45.3)	10 (29.4)		
Positive CRM (n, %)	11 (9.2)	2 (2.3)	9 (26.5)	χ^2^ = 14.284	< 0.001 ^b)^
Lymphovascular invasion (n, %)	28 (23.3)	16 (18.6)	12 (35.3)	χ^2^ = 3.794	0.051 ^b)^
Perineural invasion (n, %)	21 (17.5)	10 (11.6)	11 (32.4)	χ^2^ = 7.249	0.007 ^b)^

BMI, body mass index; cT/N stage, clinical T/N stage; MRF, mesorectal fascia; EMVI, extramural vascular invasion; APR, abdominoperineal resection; CEA, carcinoembryonic antigen; LAR: low anterior resection; CRM, circumferential resection margin; IQR, inter quartile range. “a” t-test, “b” χ^2^- test, “c” z-test, “d” Fisher’s exact test. Data are means ± standard deviation, median (IQR), or n (%) as indicated.

### The association between tumor regression response and clinicopathological characteristics

As shown in **Table 1**, patients were divided into two groups according to tumor regression response. No significant differences in age, sex, BMI, distance from the anal verge, cT stage or EMVI were noted between the two groups. Pretreatment MRI showed greater diameters of tumors before treatment [(5.54 ± 1.54) cm vs. (4.79 ± 1.57) cm, *P* = 0.034], advanced cN stage (cN2: 79.6% vs. 57.0%, *P* = 0.022) and greater MRF positivity (76.5% vs. 55.8%, *P* = 0.036) in patients in the PR group. A higher proportion of elevated CEA during TNT was observed in patients in the PR group than in those in the GR group (before treatment: 64.7% vs. 38.4%, *P* = 0.009; after chemoradiation: 55.9% vs. 10.5%, *P* < 0.001; after one cycle of CAPOX: 23.5% vs. 5.8%, *P* = 0.005; after 2 cycles of CAPOX: 23.5% vs. 4.7%, *P* = 0.006).

Regarding surgical results (**Table 1**), no significant differences in operation time, blood loss, or postoperative complications, including pelvic infection, anastomotic bleeding, anastomotic leakage, bowel obstruction and wound infection, were noted between the two groups. The rate of anal preservation was similar between the two groups. Regarding pathological results, no significant differences in lymph nodes harvested or lymphovascular invasion were noted between the two groups. Patients in the PR group had more advanced tumors, positive CRM and perineural invasion compared with those in the GR group (ypT3-4: 82.4% vs. 26.7%, *P* < 0.001; ypN1-2: 67.6% vs. 14.0%, *P* < 0.001; positive CRM: 26.5% vs. 2.3%, *P* < 0.001; perineural invasion: 32.4% vs. 11.6%, *P* = 0.007).

**Figure 2:**
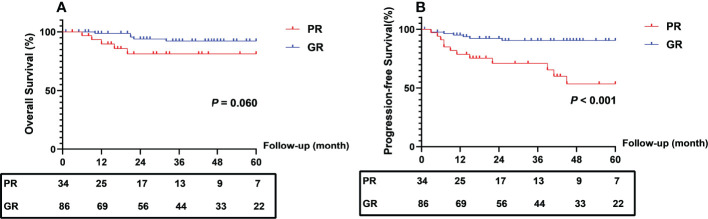
Comparison of survival outcomes between patients with different regression response. PR, poor response; GR, good response.

At the end of follow-up, 5 (14.7%) patients in the PR group died, 4 (11.8%) suffered local recurrence, and 9 (26.5%) developed distant metastasis. In the GR group, 5 (5.8%) patients in the GR group died, and 8 (9.3%) suffered distant metastasis. The PFS rate at 2 and 5 years was 70.4% and 50.0% in the PR group compared with 90.4% and 88.7% in the GR group (log-rank *P* < 0.001). The 2- and 5-year OS rates in the two groups were not significantly different (**Figure 2**).

### The association between CEA clearance after chemoradiation and clinicopathological characteristics

**Figure 3:**
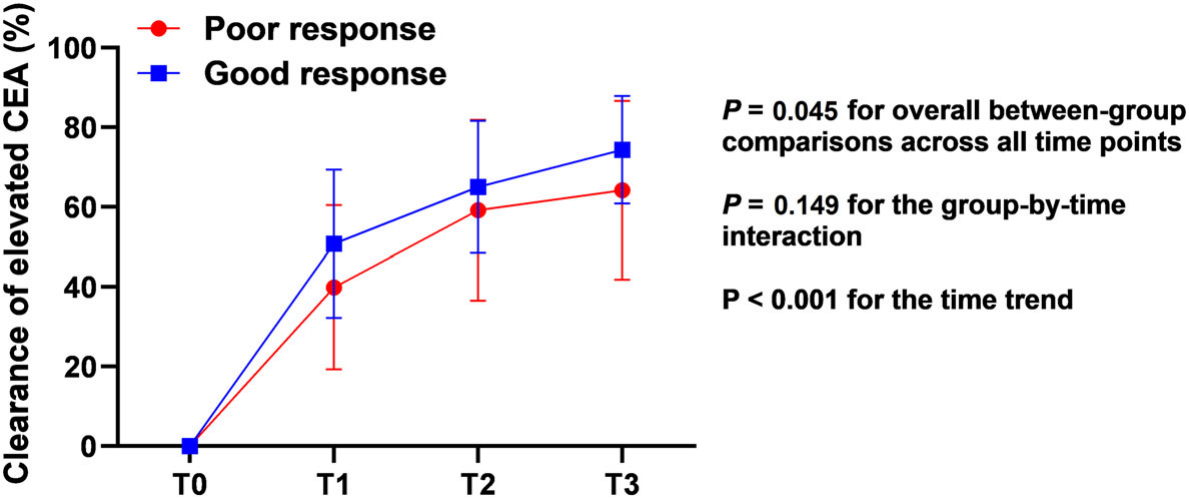
Clearance of elevated pretreatment CEA in different regression response groups during the long-period of total neoadjuvant therapy. CEA, carcinoembryonic antigen; T0, before treatment; T1, after chemoradiation; T2, after one cycle of CAPOX; T3, after 2 cycles of CAPOX.

As shown in **Table 2**, for patients with elevated pretreatment CEA, the changes in CEA during TNT were also analyzed. The clearance of elevated CEA level in the two groups during the long-course of TNT were evaluated by repeated measures analysis. There was a rapid decrease of CEA during the course (both *P* < 0.001 for time trend). Patients with good response achieved a greater clearance rate of CEA level than those with poor response (*P* = 0.045) (**Figure 3**). Despite the lack of significant differences in CEA after the completion of TNT, fewer patients in the PR group obtained normal CEA after chemoradiation (13.6% vs. 72.7%, *P* < 0.001). Besides, more patients’ CEA levels in the GR group decreased by greater than 50% after chemoradiation when compared with that in the PR group (60.6% vs. 18.2%, *P* = 0.002).

**Table 2:** Changes of carcinoembryonic antigen in patients with elevated carcinoembryonic antigen during total neoadjuvant therapy.

Variables	Good response (n = 33)	Poor response (n = 22)	Statistics	*P* value
CEA after chemoradiation			χ^2^ = 18.442	< 0.001 ^b)^
< 5.0 ng/ml	24 (72.7)	3 (13.6)		
≥ 5.0 ng/ml	9 (27.3)	19 (86.4)		
CEA after one cycle of CAPOX			χ^2^ = 1.981	0.070 ^b)^
< 5.0 ng/ml	28 (84.8)	14 (63.6)		
≥ 5.0 ng/ml	5 (15.2)	8 (36.4)		
CEA after 2 cycles of CAPOX			χ^2^ = 3.238	0.072 ^b)^
< 5.0 ng/ml	29 (87.9)	14 (63.6)		
≥ 5.0 ng/ml	4 (12.1)	8 (36.4)		
The clearance of elevated CEA after chemoradiation			χ^2^ = 9.659	0.002 ^b)^
< 50%	13 (39.4)	18 (81.8)		
≥ 50%	20 (60.6)	4 (18.2)		
The clearance of elevated CEA after one cycle of CAPOX			χ^2^ = 0.783	0.376 ^b)^
< 50%	7 (21.2)	7 (31.8)		
≥ 50%	26 (78.8)	15 (68.2)		
The clearance of elevated CEA after 2 cycles of CAPOX			χ^2^ = 1.030	0.310 ^b)^
< 50%	3 (9.1)	5 (22.7)		
≥ 50%	30 (90.9)	17 (77.3)		

CEA, carcinoembryonic antigen. “b” χ^2^- test.

According to the CEA clearance pattern after chemoradiation, patients were divided into two groups. Group A comprised patients with normal pretreatment CEA, or those of whom CEA was greater than 5 ng/ml before treatment but decreased by greater than 50% after chemoradiation. Patients in Group B obtained an elevated pretreatment CEA and decreased by less than 50% after chemoradiation. Groups A and B comprised 99 and 21 patients, respectively.

As shown in **Table 3**, there were no significant differences regarding age, sex, BMI, distance from the anal verge, cT/N stage MRF or EMVI between two groups. No significant differences in operation time, blood loss, or postoperative complications were noted between the two groups. Regarding pathological results, no significant differences in lymph nodes harvested, pCR, perineural invasion or lymphovascular invasion were noted between the two groups. Patients in Group B had more advanced tumors and positive CRM compared with those in Group A (ypT3-4: 71.4% vs. 36.4%, *P* < 0.001; ypN1-2: 61.9% vs. 22.2%, *P* < 0.001; positive CRM: 28.6% vs. 5.1%, *P* = 0.003). Group B was more likely to achieve poor tumor response to TNT than Group A (76.2% vs. 18.2%, *P* < 0.001).

**Table 3:** Comparison of clinical and pathological characteristics of patients with locally advanced rectal cancer following total neoadjuvant therapy stratified by the clearance in the carcinoembryonic antigen level after chemoradiation.

Characteristics	Group A (n = 99)	Group B (n = 21)	Statistics	*P* value
Age (x ± SD), years	60.50 ± 10.56	57.50 ± 9.11	t = 1.228	0.222 ^a)^
Gender (n, %)			χ^2^ = 0.103	0.748 ^b)^
Male	77 (77.8)	17 (81.0)		
Female	22 (22.2)	4 (19.0)		
BMI (x ± SD), kg/m^2^	24.05 ± 3.32	25.33 ± 3.75	t = -1.630	0.106 ^a)^
Distance from anal verge (n, %)			χ^2^ = 2.049	0.152 ^b)^
≤ 5 cm	50 (50.5)	7 (33.3)		
> 5 cm	49 (49.5)	14 (66.7)		
Tumor diameters before treatment (x ± SD), cm	4.27 ± 2.22	5.24 ± 2.21	t = -1.878	0.063 ^a)^
cT stage before treatment (n, %)			χ^2^ = 0.481	0.488 ^b)^
cT2-3	73 (73.7)	17 (81.0)		
cT4	26 (26.3)	4 (19.0)		
cN stage before treatment (n, %)			χ^2^ = 0.718	0.397 ^b)^
cN0-1	38 (38.4)	6 (28.6)		
cN2	61 (61.6)	15 (71.4)		
MRF before treatment (n, %)			χ^2^ = 1.026	0.311 ^b)^
Positive	59 (59.6)	15 (71.4)		
Negative	40 (40.4)	6 (28.6)		
EMVI before treatment (n, %)			χ^2^ = 0.536	0.464 ^b)^
Positive	43 (43.9)	7 (35.0)		
Negative	55 (56.1)	13 (65.0)		
Missing	1	1		
Grade 3/4 adverse events [n (%)]				
Total	23 (23.2)	6 (28.6)	χ^2^ = 0.269	0.604 ^b)^
Neutropenia	12 (12.1)	3 (14.3)	χ^2^ = 0.000	1.000 ^b)^
Anemia	5 (5.1)	2 (9.5)	χ^2^ = 0.079	0.778 ^b)^
Thrombocytopenia	7 (7.1)	2 (9.5)	χ^2^ =0.000	1.000 ^b)^
Diarrhea	4 (4.0)	1 (4.8)		1.000 ^d)^
Vomiting	0 (0)	1 (4.8)	–	0.175 ^d)^
Radiation dermatitis	2 (2.0)	2 (9.5)	–	0.141 ^d)^
Rectal pain	5 (5.1)	2 (9.5)	χ^2^ = 0.079	0.778 ^b)^
Operation approaches (n, %)			χ^2^ = 2.876	0.237 ^b)^
LAR	74 (74.7)	12 (57.1)		
APR	20 (20.2)	8 (38.1)		
Hartmann	5 (5.1)	1 (4.8)		
Operation time (median, IQR), min	240 (202-300)	198 (156-300)	Z = -1.792	0.073 ^c)^
Blood loss (median, IQR), ml	100 (80-200)	100 (65-225)	Z = -0.352	0.725 ^c)^
Complications (n, %)				
Pelvic infection	3 (3.0)	0 (0)	–	1.000 ^d)^
Anastomotic bleeding	1 (1.0)	1 (4.8)	–	0.321 ^d)^
Anastomotic leakage	5 (5.1)	2 (9.5)	χ^2^ = 0.079	0.778 ^b)^
Bowel obstruction	5 (5.1)	2 (14.3)	χ^2^ = 2.375	0.123 ^b)^
ypT stage (n, %)			χ^2^ = 8.717	0.003 ^b)^
ypT0~2	63 (63.6)	6 (28.6)		
ypT3~4	36 (36.4)	15 (71.4)		
ypN stage (n, %)			χ^2^ = 13.205	< 0.001 ^b)^
ypN0	77 (77.8)	8 (38.1)		
ypN1~2	22 (22.2)	13 (61.9)		
Lymph nodes harvested (n, %)			χ^2^ = 0.593	0.441 ^b)^
≥ 12	57 (57.6)	14 (66.7)		
< 12	42 (42.4)	7 (33.3)		
Tumor regression (n, %)			χ^2^ = 28.711	< 0.001 ^b)^
Good (TRG 3-4)	81 (81.8)	5 (23.8)		
Poor (TRG1-2)	18 (18.2)	16 (76.2)		
pCR (n, %)	25 (25.3)	2 (9.5)	χ^2^ = 1.639	0.201 ^b)^
Positive CRM (n, %)	5 (5.1)	6 (28.6)	χ^2^ = 8.860	0.003 ^b)^
Lymphovascular invasion (n, %)	20 (20.2)	8 (38.1)	χ^2^ = 2.181	0.140 ^b)^
Perineural invasion (n, %)	14 (14.1)	7 (33.3)	χ^2^ = 3.191	0.074 ^b)^

BMI, body mass index; cT/N stage, clinical T/N stage; MRF, mesorectal fascia; EMVI, extramural vascular invasion; APR, abdominoperineal resection; CEA, carcinoembryonic antigen; LAR, low anterior resection; CRM, circumferential resection margin; IQR, inter quartile range. “a” t-test, “b” χ^2^- test, “c” z-test, “d” Fisher’s exact test. Data are means ± standard deviation, median (IQR), or n (%) as indicated.

**Figure 4:**
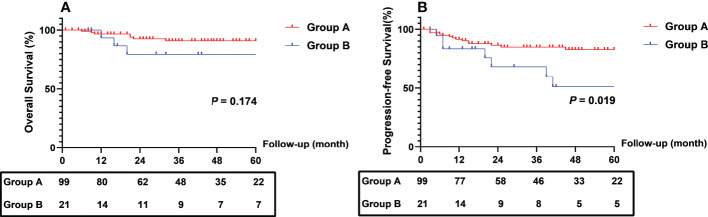
Comparison of survival outcomes between patients with different different CEA clearance pattern. CEA, carcinoembryonic antigen.

The Kaplan-Meier curve of CEA clearance pattern after chemoradiation for OS and PFS are shown in **Figure 4**. Patients in Group B had worse 2- (68.2% vs. 86.4%) and 5-year (51.1% vs. 82.7%) PFS than those in Group A (log-rank *P* = 0.019). The 2- and 5-year OS rates in the two groups were not significantly different.

### Multivariable analysis and ROC curves for risk factors affecting regression response to TNT

Univariate analysis revealed that tumor diameter ≥ 5.0 cm, cN2, MRF positivity before treatment, CEA before treatment ≥ 5.0 ng/ml, CEA after chemoradiation ≥ 5.0 ng/ml, CEA after one cycle of CAPOX ≥ 5.0 ng/ml, CEA after 2 cycles of CAPOX ≥ 5.0 ng/ml and CEA clearance pattern Group B after chemoradiation were risk factors for poor regression response. Logistic multivariable analysis including the results in all study patients identified tumor diameter before treatment ≥ 5.0 cm (HR 6.943, 95% CI 2.250-21.428, *P* = 0.001), cN2 (HR 5.054, 95% CI 1.553-16.448, *P* = 0.007) and CEA clearance pattern Group B after chemoradiation (HR 8.435, 95% CI 1.062-66.992, *P* = 0.044) as independent risk factors for poor regression response (**Table 4**).

**Table 4:** Univariate and logistic multivariate analyses for predicting tumor regression response of patients with locally advanced rectal cancer following total neoadjuvant therapy.

Variables (n, %)	N	Poor response	Univariate analyses		Logistic multivariate analyses	
			HR (95% CI)	*P* value	HR (95% CI)	*P* value
Age				0.235		
< 60 years	49	11	1.000			
≥ 60 years	71	23	1.655 (0.718-3.815)			
Gender				0.857		
Male	94	27	1.000			
Female	26	7	0.914 (0.345-2.424)			
BMI				0.725		
< 24.0 kg/m2	56	15	1.000			
≥ 24.0 kg/m2	64	19	1.154 (0.519-2.564)			
Distance from anal verge				0.951		
> 5 cm	63	18	1.000			
≤ 5 cm	57	16	1.025 (0.463-2.271)			
Tumor diameters before treatment				0.003		0.001
< 5.0 cm	64	10	1.000		1.000	
≥ 5.0 cm	54	22	3.644 (1.531-8.669)		6.943 (2.250-21.428)	
Missing	2					
cT stage				0.815		
cT2-3	90	26	1.000			
cT4	30	8	0.895 (0.354-2.266)			
cN stage				0.025		0.007
cN0-1	44	7	1.000		1.000	
cN2	76	27	2.913 (1.144-7.415)		5.054 (1.553-16.448)	
MRF				0.039		0.188
Negative	46	8	1.000		1.000	
Positive	74	26	2.573 (1.047-6.325)		2.137 (0.689-6.626)	
EMVI				0.513		
Negative	68	18	1.000			
Positive	50	16	1.307 (0.586-2.915)			
Missing	2					
CEA before treatment				0.010		0.101
< 5.0 ng/ml	65	12	1.000		1.000	
≥ 5.0 ng/ml	55	22	2.944 (1.288-6.731)		0.290 (0.066-1.271)	
CEA after chemoradiation				< 0.001		0.086
< 5.0 ng/ml	92	15	1.000		1.000	
≥ 5.0 ng/ml	28	19	10.837 (4.121-28.501)		6.182 (0.772-49.488)	
CEA after one cycle of CAPOX				0.009		0.515
< 5.0 ng/ml	107	26	1.000		1.000	
≥ 5.0 ng/ml	13	8	4.985 (1.499-16.575)		0.354 (0.016-8.057)	
CEA after 2 cycles of CAPOX				0.022		0.620
< 5.0 ng/ml	108	26	1.000		1.000	
≥ 5.0 ng/ml	12	8	4.200 (1.231-14.333)		1.752 (0.191-16.037)	
CEA clearance pattern				< 0.001		0.044
Group A	99	18	1.000		1.000	
Group B	21	16	14.40 (4.668-44.426)		8.435 (1.062-66.992)	

BMI, body mass index; cT/N stage, clinical T/N stage; MRF, mesorectal fascia; EMVI, extramural vascular invasion; CEA, carcinoembryonic antigen.

**Figure 5:**
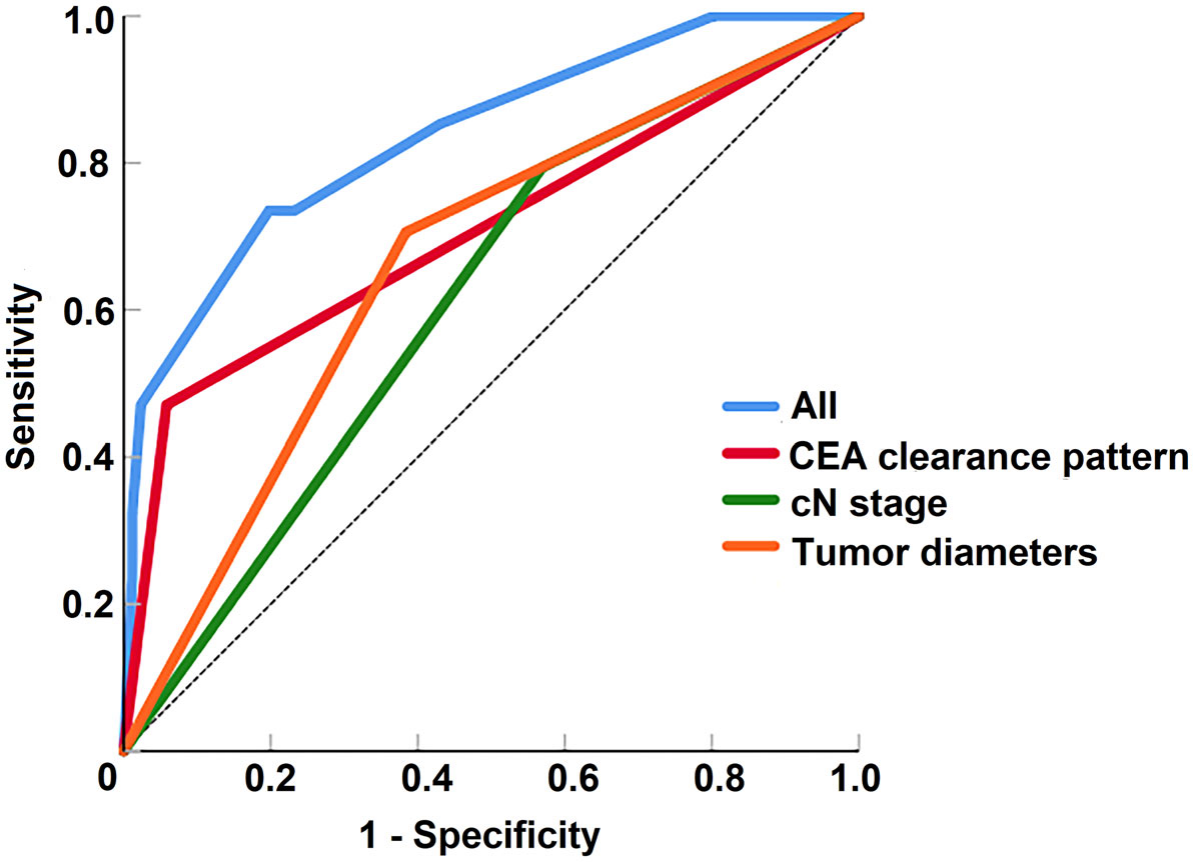
The receiver operating characteristic curves of risk factors for the prediction of tumor regression response to total neoadjuvant therapy.

According to the multivariable analysis, the ROC curves for poor regression response to TNT were performed. The ability of discrimination of the CEA clearance pattern after chemoradiation, tumor diameter and cN stage, as assessed by AUC, was 0.706 (95%CI 0.591-0.821), 0.661 (95%CI 0.554-0.769) and 0.612 (95%CI 0.504-0.720). The AUC of all of these risk factors was 0.836 (95%CI 0.754-0.919) (**Figure 5**).

## Discussion

TNT has been applied as the optional treatment for LARC patients (8); however, patients differ in their tumor regression response (10, 13). Early identification of patients who might not benefit from TNT and alterations in therapeutic approaches seemed to exhibit improved prognosis. In this single-institution retrospective study, we collected the clinical and pathological characteristics during the TNT course and further explore factors that might predict a tumor regression response during TNT.

In the current study, the most common AEs include neutropenia (12.5%), thrombocytopenia (7.5%), anemia (5.8%) and rectal pain (5.8%). No grade 4 or serious adverse events were observed. In addition, 27 (22.5%) patients achieved pCR in this study, which was consistent with the pCR rate reported by other studies (10, 11). Therefore, the TNT regimens in our center were considered safe and effective.

In this study, the PFS of patients in the PR group was inferior to that of GR patients. Other studies also reported that patients with pCR were likely to achieve better survival outcomes, indicating that relatively poor regression response was associated with worse survival outcomes (18, 19). The worse survival outcomes might be attributed to advanced ypT/N stage, positive CRM and lymphovascular invasion in patients in the PR group. R0 resection might be difficult to achieve for tumors with slight or no regression, resulting in a positive CRM (20). In our study, the distant metastasis and local recurrence rates of patients with positive CRM were as high as 25% and 37.5%, respectively. As reported in the RAPIDO trial (10), the increased number of cycles of chemotherapy and extended time interval between radiotherapy and surgery might not benefit patients with poor response to TNT. These patients might actually progress during preoperative treatment, making it important to identify predictive factors for poor regression response to TNT.

In the present study, patients with larger and node-positive tumors were more likely to have a poor regression response, and logistic multivariable analysis showed that tumor diameter ≥ 5.0 cm and cN2 before treatment were independent risk factors leading to poor response. Consistent with the findings of our study, the retrospective study by Chapman et al. (13) also identified that larger and node-positive tumors were predictive of incomplete regression response. Jankowski et al. (21) showed that the clinical complete regression rates in rectal patients with tumor diameters of 1-3 cm, 4 cm, 5-6 cm and 7 cm were 36.5%, 22.9%, 7.3% and 3.4%, respectively (*P* < 0.001). The author considered that the tumor diameter might be a clinical predictor of tumor response. In the study conducted by Yoon et al. (22) with a total of 351 patients, tumor regression was observed in 103 patients (29.3%), and complete regression was observed in 51 patients (14.5%). Multivariable analysis showed that cN1-2 was an independent poor regression response (*P* = 0.044). A larger tumor diameter before treatment and cN2 might be indicative of a biologically more aggressive tumor.

The surveillance of CEA levels during TNT might be feasible for the identification of poor response. In the present study, we found that patients who achieved a poor response were more likely to have an elevated CEA level at each time point. Serum CEA levels are widely used as a tumor marker in patients with colorectal cancer and have been reported to predict the response to neoadjuvant therapy (23, 24). Cheong et al. (14) found that CEA levels before and after neoadjuvant treatment were both important risk factors for pCR and good response (all *P* < 0.05). Chapman et al. (13) also suggested that pretreatment CEA levels may predict the response to TNT treatment. The change in CEA levels during the whole TNT course might also predict tumor regression after TNT. In the present study, for those with elevated pretreatment CEA, more patients whose CEA decreased by greater than 50% after chemoradiation had a good response to TNT. Besides, repeated measures analysis showed that patients with good response achieved a greater clearance rate of elevated CEA level than those with poor response. Hu et al. (25) also demonstrated that CEA clearance was an independent predictor of tumor response to neoadjuvant treatment. CEA clearance suggested a decrease in biological activity (14), which also showed a response to TNT treatment.

The CEA level after chemoradiation might have important implications for the response to TNT. Among those with elevated pretreatment CEA in this study, more patients who finally achieved a good regression response obtained a normal CEA after chemoradiation than those with poor response. According to CEA clearance after chemoradiation, we divided patients into two groups, and found that patients who obtained an elevated pretreatment CEA and decreased by less than 50% after chemoradiation were associated with worse tumor regression response and survival outcomes. We considered that the abnormal CEA level after chemoradiotherapy might suggest a poor response to TNT treatment, which might be associated with the completion of IMRT (26). Radiotherapy was able to kill cancer cells through high doses of radiation by damaging DNA directly or by creating free radicals within the cells, thereby destroying the tumor tissues (27). As a radiosensitizer, the administration of radiotherapy following CAPOX during TNT significantly boosted the effectiveness of preoperative IMRT and resulted in the early eradication of micrometastases (28–30). Based on these findings, the completion of chemoradiation can be regarded as an essential time point of the TNT course, which might determine patients’ regression response.

In the current study, logistic multivariable analysis showed CEA clearance pattern after chemoradiation, tumor diameter and cN stage were risk factors affecting poor response to TNT treatment. The ROC curves also confirmed that these factors could better assess the regression response of patients who received TNT treatment in this study. Therefore, we considered that active surveillance in circulating tumor DNA or tumor-related characteristics like tumor diameter and stage during the long course of TNT treatment could help early identification of patients with poor regression response. In this study, we only performed analysis regarding CEA, and further research is needed to develop a accurate prediction model.

As previously described, greater than 20% of patients are resistant to treatment or develop progressive disease. As mentioned in the RAPIDO trial (10), early identification of poor regression response could enable alterations in therapeutic approach. For patients at high risk for poor response to TNT, targeted therapy and immunotherapy might be considered optional approaches. Recent studies have suggested favorable pathological outcomes following neoadjuvant chemoradiotherapy plus targeted therapy (31, 32). Immunotherapies, such as programmed cell death-1 or programmed cell death-ligand 1 inhibitors, also demonstrate encouraging clinical benefits including response rate, and the immune checkpoint represent a useful target to enhance tumor regression (33, 34). Further investigation in a large cohort study is needed to improve prognosis in patients who are resistant to TNT.

Several limitations of our study deserve mention. This was a single-center study that involved a relatively small number of patients, which limited our ability to investigate more risk factors and establish prediction models of regression in response to TNT treatment. Additionally, genetic tests regarding mismatch repair and microsatellite instability, which might affect the biological behavior of tumor cells, were lacking in our study. Moreover, MRI assessment was only performed before and after the TNT course, and patients in this study did not receive MRI restaging during the TNT course. Although MRI was recommended by the NCCN guidelines for the staging of rectal cancer, it was demonstrated that MRI cannot accurately assess tumor response to preoperative chemoradiotherapy (35). It has been reported that diffusion-weighted and gadolinium-enhanced MRI yields better diagnostic accuracy in the evaluation of response to neoadjuvant chemoradiotherapy in LARC patients, and further study will investigate their value in the prediction of response to TNT treatment (36–38). Finally, our inclusion of patients exclusively treated with TNT and consolidation chemotherapy is likely not reflective of all the current TNT approaches to rectal cancer. More trials are needed to further demonstrate the role of active surveillance during the TNT course in the early identification of poor response.

## Conclusion

In LARC patients who are treated with the TNT strategy, approximately one-fourth will achieve a poor regression response. Larger tumors, cN2 tumors before treatment and elevated CEA levels were considered predictive features of a poor response. Active surveillance of CEA levels during TNT might be feasible for the early identification of a poor response. CEA levels after chemoradiation might have important implications for the response to TNT. Large-scale, prospective experiments are needed to further and accurately identify tumor regression response and explore the optional administration strategy to treat patients at high risk for poor response to TNT.

## Data availability statement

The raw data supporting the conclusions of this article will be made available by the authors, without undue reservation.

## Ethics statement

The studies involving human participants were reviewed and approved by the local Ethics Committee of Beijing Chaoyang Hospital. The patients/participants provided their written informed consent to participate in this study.

## Author contributions

Study conception and design: ZZ, GW, HQ, HZ, ZW, JH. Acquisition of data: HZ, GL, KC. Analysis and interpretation of data: HZ, GL, KC. Drafting of manuscript: HZ, GL, KC. Final approval: ZZ, GW, ZW, JH, HZ, GL, KC, HQ. Critical revision: JH, ZW, ZZ, GW, HQ. All authors contributed to the article and approved the submitted version.

## Acknowledgments

We thank American Journal Experts (https://www.aje.cn) for editing this manuscript.

## Conflict of interest

The authors declare that the research was conducted in the absence of any commercial or financial relationships that could be construed as a potential conflict of interest.

## Publisher’s note

All claims expressed in this article are solely those of the authors and do not necessarily represent those of their affiliated organizations, or those of the publisher, the editors and the reviewers. Any product that may be evaluated in this article, or claim that may be made by its manufacturer, is not guaranteed or endorsed by the publisher.
